# Inhibition of Dehydration-Induced Water Intake by Glucocorticoids Is Associated with Activation of Hypothalamic Natriuretic Peptide Receptor-A in Rat

**DOI:** 10.1371/journal.pone.0015607

**Published:** 2010-12-20

**Authors:** Chao Liu, Jing Guan, Yunxiao Kang, Heming Xiu, Ying Chen, Bao Deng, Kunshen Liu

**Affiliations:** 1 Heart Center, The First Hospital of Hebei Medical University, Hebei Medical University, Shijiazhuang, People's Republic of China; 2 Stomatology Division, Hebei General Hospital, Shijiazhuang, People's Republic of China; 3 Department of Neurobiology, Hebei Medical University, Shijiazhuang, People's Republic of China; 4 Central Laboratory, The First Hospital of Hebei Medical University, Hebei Medical University, Shijiazhuang, People's Republic of China; Georgia State University, United States of America

## Abstract

Atrial natriuretic peptide (ANP) provides a potent defense mechanism against volume overload in mammals. Its primary receptor, natriuretic peptide receptor-A (NPR-A), is localized mostly in the kidney, but also is found in hypothalamic areas involved in body fluid volume regulation. Acute glucocorticoid administration produces potent diuresis and natriuresis, possibly by acting in the renal natriuretic peptide system. However, chronic glucocorticoid administration attenuates renal water and sodium excretion. The precise mechanism underlying this paradoxical phenomenon is unclear. We assume that chronic glucocorticoid administration may activate natriuretic peptide system in hypothalamus, and cause volume depletion by inhibiting dehydration-induced water intake. Volume depletion, in turn, compromises renal water excretion. To test this postulation, we determined the effect of dexamethasone on dehydration-induced water intake and assessed the expression of NPR-A in the hypothalamus. The rats were deprived of water for 24 hours to have dehydrated status. Prior to free access to water, the water-deprived rats were pretreated with dexamethasone or vehicle. Urinary volume and water intake were monitored. We found that dexamethasone pretreatment not only produced potent diuresis, but dramatically inhibited the dehydration-induced water intake. Western blotting analysis showed the expression of NPR-A in the hypothalamus was dramatically upregulated by dexamethasone. Consequently, cyclic guanosine monophosphate (the second messenger for the ANP) content in the hypothalamus was remarkably increased. The inhibitory effect of dexamethasone on water intake presented in a time- and dose-dependent manner, which emerged at least after 18-hour dexamethasone pretreatment. This effect was glucocorticoid receptor (GR) mediated and was abolished by GR antagonist RU486. These results indicated a possible physiologic role for glucocorticoids in the hypothalamic control of water intake and revealed that the glucocorticoids can act centrally, as well as peripherally, to assist in the normalization of extracellular fluid volume.

## Introduction

Atrial natriuretic peptide (ANP), a peptide hormone secreted by the heart in response to volume expansion, revealed a homeostatic mechanism that counterbalanced the salt- and water-retaining actions of the renin–angiotensin–aldosterone system that predominates in terrestrial animals. The primary receptor of ANP, natriuretic peptide receptor-A (NPR-A) is localized mostly in the kidney, but also is found in hypothalamic area involved in body fluid volume regulation [1], [2], [3]. Renal NPR-A activation induces potent diuresis and natriuresis, whereas hypothalamic NPR-A activation inhibits dehydration-induced water intake [4], [5]. Previous evidences showed that glucocorticoids could upregulate ANP mRNA expression in the atrial myocytes and increase the ANP levels in the circulation [6], [7], [8]. Recent evidences further showed that glucocorticoids could upregulate NPR-A expression in the kidney [9]. It is postulated that acute glucocorticoid administration produces potent diuresis and natriuresis, possibly acting in the natriuretic peptide system in the kidney [6], [7], [8], [9], [10]. However, it cannot account for the fact that chronic glucocorticoid administration attenuates renal water and sodium excretion in intact rats [11], [12]. The precise mechanism underlying these paradoxical finding is unclear. Thus, we raised a hypothesis that chronic glucocorticoid administration may activate natriuretic peptide system in hypothalamus, and cause volume depletion by inhibiting dehydration-induced water intake. Volume depletion, in turn, compromises renal water and sodium excretion. To test the hypothesis, we carried out this study.

## Results

### The effect of 6-hour Dex pretreatment on urinary volume and water intake

Thirty male Wistar rats were deprived of water for 24 hours to have dehydration status. Then, tap water was given, and water intake was monitored over 12 hours. The water-deprived rats were randomized to receive vehicle, low dose of dexamethasone sodium phosphate (Dex, 0.1 mg/kg) or high dose of Dex (1.0 mg/kg) 6 hours prior to free access to water.

**Figure 1:**
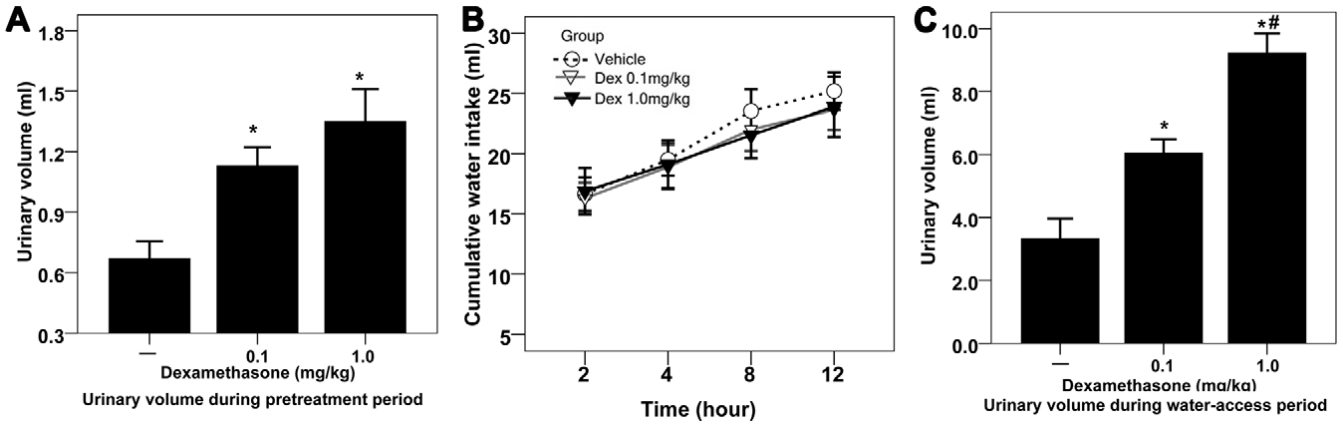
The effect of 6-hour Dex pretreatment on urinary volume and water intake. [**A**] Effect of Dex pretreatment on urinary volume during 6-hour pretreatment period; *<0.01 compared with rats treated with vehicle. [**B**] Effect of Dex pretreatment on cumulative water intake during water-access period in the dehydrated rats; n = 10 for each group; data were analyzed by two-way repeated measures ANOVA. [**C**] Effect of Dex pretreatment on urinary volume during water-access period; *<0.01 compared with rats treated with vehicle; # <0.01 compared with rats treated with low dose of Dex.

In the 6-hour pretreatment period, Dex pretreatment doubled renal water excretion in the water-deprived rats (Fig. 1A). But, 6-hour Dex pretreatment did not affect subsequent dehydration-induced water intake over 12-hour water-access period (Fig. 1B). Of note, Dex-pretreated rats produced a potent, dose-dependent diuresis during 12-hour water-access period (Fig. 1C). In view of the fact that glucocorticoids produced potent diuresis without affecting water intake, relative volume depletion, inevitably, occurred.

### The effect of 18-hour Dex pretreatment on urinary volume and water intake

The 24-hour water-deprived rats were randomized to receive vehicle, low dose of Dex or high dose of Dex 18 hours prior to free access to water.

**Figure 2:**
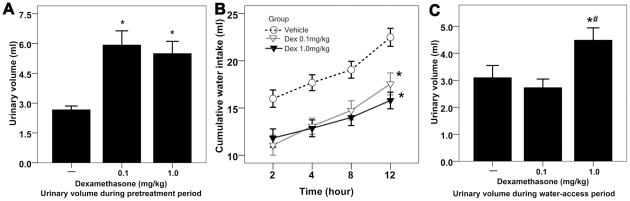
The effect of 18-hour Dex pretreatment on urinary volume and water intake. [**A**] Effect of Dex pretreatment on urinary volume during 18-hour pretreatment period; *<0.01 compared with rats treated with vehicle. [**B**] Effect of Dex pretreatment on cumulative water intake during water-access period in the dehydrated rats; n = 10 for each group; data were analyzed by two-way repeated measures ANOVA; *<0.01 compared with rats treated with vehicle. [**C**] Effect of Dex pretreatment on urinary volume during water-access period; *<0.01 compared with rats treated with vehicle; # <0.01 compared with rats treated with low dose of Dex.

During the 18-hour pretreatment period, Dex pretreatment doubled renal water excretion in the water-deprived rats (Fig. 2A). The 18-hour Dex pretreatment dramatically inhibited the dehydration-induced water intake over 12-hour water-access period, but there was no difference in water intake between two Dex-pretreated groups (Fig. 2B). Even though, the water-deprived rats pretreated with large dose of Dex produced a remarkable diuresis in 12-hour water-access period (Fig. 2C).

### The effect of 24-hour Dex pretreatment on urinary volume and water intake

**Figure 3:**
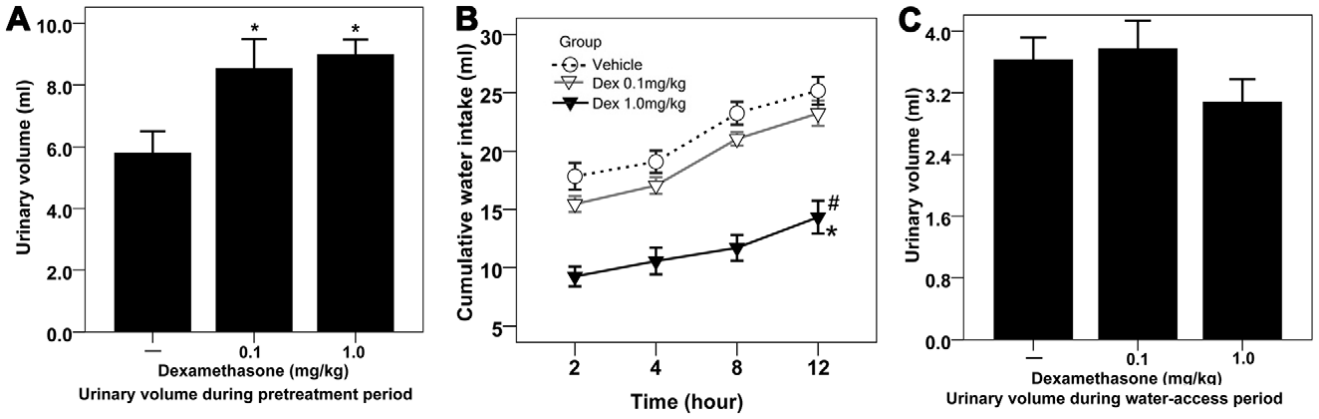
The effect of 24-hour Dex pretreatment on urinary volume and water intake. [**A**] Effect of Dex pretreatment on urinary volume during 24-hour pretreatment period; *<0.01 compared with rats treated with vehicle. [**B**] Effect of Dex pretreatment on cumulative water intake during water-access period in the dehydrated rats; n = 10 for each group; data were analyzed by two-way repeated measures ANOVA; *<0.01 compared with rats treated with vehicle; # <0.01 compared with rats treated with low dose of Dex. [**C**] Effect of Dex pretreatment on urinary volume during water-access period.

As pretreatment prolonged to 24 hours, the water intake in rats treated with large dose of Dex was dramatically decreased compared with the water intake in the rats pretreated with vehicle or low dose of Dex. The rats pretreated with low dose of Dex drank less water compared with vehicle pretreated rats, but there was no statistical difference in water intake between two groups (Fig. 3B). There was a slight, but not a statistically significant, reduction in urinary volume in the rats pretreated with large dose of Dex (Fig. 3C).

Collectively, Dex not only produced a potent diuresis but inhibited dehydration-induced water intake. The effect was both time- and dose- dependent. Of note, as the inhibitory effect of glucocorticoids on drinking emerges, the diuretic effects of glucocorticoids were attenuated.

### The effect of Dex on hypothalamic NPR-A expression and cGMP content

Earlier observations showed that infusion of ANP into the third cerebral ventricle of conscious rats inhibited dehydration- or angiotensin II (AII)-induced water intake, and salt depletion-induced salt appetite in conscious rats, supporting an important role of natriuretic peptide system in the central control of extracellular fluid volume and electrolyte composition [1], [13].

**Figure 4:**
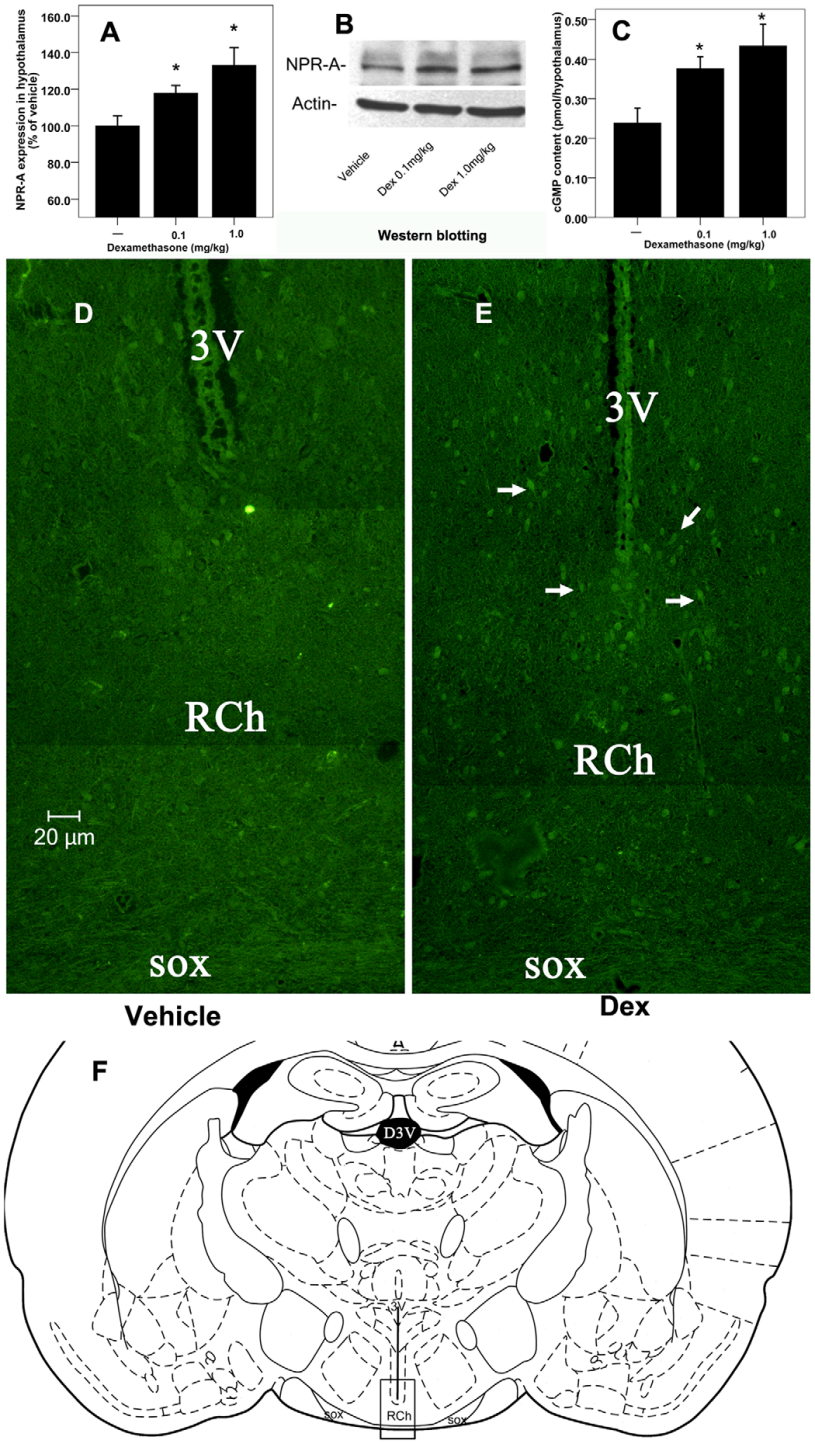
The effect of Dex on hypothalamic NPR-A expression and cGMP content in the water-deprived rats. [**A**], [**B**] Effect of Dex pretreatment on hypothalamic NPR-A expression in the water-deprived rats. [**C**] Effect of Dex on hypothalamic cGMP content in the water-deprived rats. n = 8 for each group. *<0.05 compared with vehicle. [**D**], [**E**] NPR-A positive neurons visualized by immunofluorescence in the hypothalamus (white arrows denote increased NPR-A positive neurons) in the water-deprived rats. Abbreviations: RCh, retrochiasmatic nucleus; sox, Supraoptic decussation; 3V, third ventricle. [**F**] Schematic drawing of a coronal section of the rat brain (from ref. [37]) at the level shown in [D] and [E]. The area defined by the square is shown in [D] and [E].

To determine the effect of Dex on hypothalamic NPR-A expression, we assessed its expression using western blotting analysis. It showed that 24-hour Dex pretreatment remarkably increased hypothalamic NPR-A expression in the water-deprived rats, compared with vehicle pretreatment. However, there was no statistical difference in hypothalamic NPR-A expression between Dex-pretreated rats (Fig. 4A, B). Consistent with the western blotting analysis, NPR-A positive neurons in the hypothalamus visualized by immunofluorescence were dramatically increased by Dex (Fig. 4D, E).

Hypothalamic cyclic guanosine monophosphate (cGMP, the second messenger for the ANP) content serves as a useful biological marker for the activity of NPR-A in vivo. There was a dramatic increase in hypothalamic cGMP content in the Dex-pretreated rats. However, the hypothalamic cGMP content in the rats pretreated with high dose of Dex was slightly, but not statistically significantly, higher than that in rats pretreated with low dose of Dex for 24 hours (Fig. 4C).

**Figure 5:**
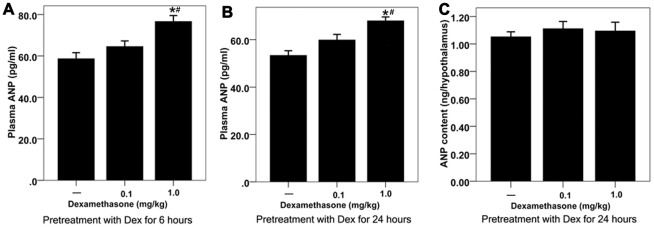
The effect of Dex pretreatment on plasma ANP and hypothalamic ANP content in the water-deprived rats. [**A**] Effect of 6-hour Dex pretreatment on plasma ANP levels in water-deprived rats; *<0.01 compared with rats treated with vehicle; # <0.01 compared with rats treated with low dose of Dex. [**B**] Effect of 24-hour Dex pretreatment on plasma ANP levels in water-deprived rats; *<0.01 compared with rats treated with vehicle; # <0.01 compared with rats treated with low dose of Dex. [**C**] Effect of 24-hour Dex pretreatment on hypothalamic ANP contents in water-deprived rats; n = 10 for each group.

The water-deprived rats pretreated with large dose of Dex but not low dose of Dex had a slightly, but nevertheless, statistically higher ANP levels than that in vehicle pretreated rats both in 6- and 24-hour Dex pretreated rats (Fig. 5A, B). However, the change in ANP levels was quantitively smaller, which cannot account for the inhibitory effect on water intake induced by Dex. In addition, Dex had no effect on ANP levels in the hypothalamus (Fig. 5C), which was consistent with previous findings [14], [15], [16]. Therefore, the upregulation of hypothalamic NPR-A expression by Dex could contribute to the NPR-A activation in the hypothalamus.

### Reversal of the effects of Dex on body fluid control by RU486

**Figure 6:**
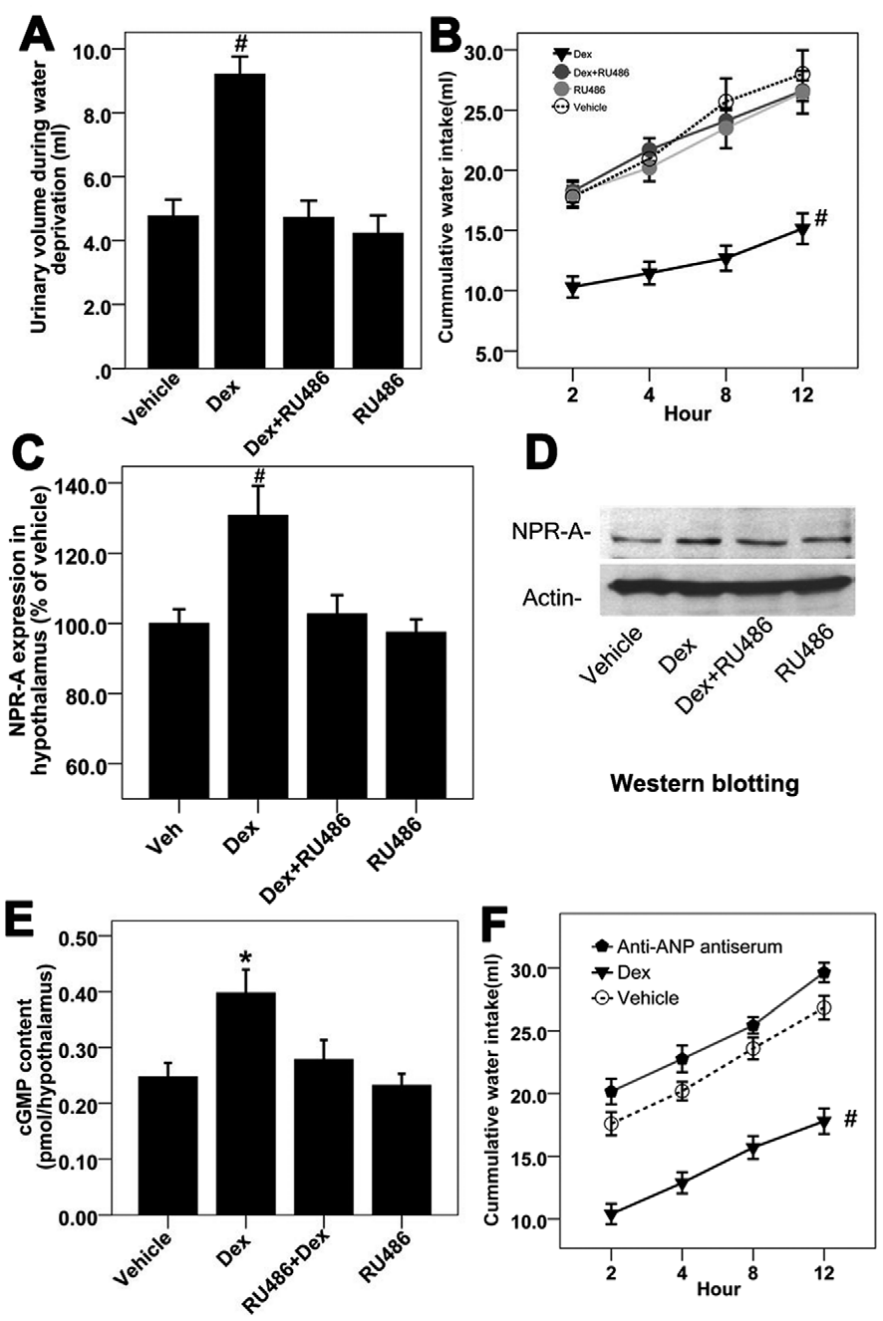
Reversal of the effects of Dex on body fluid metabolism by RU486 in the water-deprived rats. [**A**] RU486 reversed the effect of Dex on urinary volume in the water-deprived rats. #<0.01 compared with rats treated with vehicle. [**B**] RU486 reversed the effect of Dex on water intake over 12-hour water-access period in the dehydrated rats; n = 10 for each group; data were analyzed by two-way repeated measures ANOVA; # <0.01 compared with rats treated with vehicle. [**C**], [**D**], [**E**] RU486 reversed the effect of Dex on hypothalamic NPR-A expression and cGMP production in the water-deprived rats; n = 8 for each group; *<0.05 compared with rats treated with vehicle; # <0.01 compared with rats treated with vehicle. [**F**] Rabbit anit-hANP antiserum blocked the inhibitory effects of Dex on water intake in water-deprived rats; n = 5–6 in each group; vehicle  =  normal rabbit serum; data were analyzed by two-way repeated measures ANOVA; # <0.01 compared with rats treated with vehicle.

With the use of RU486, it is possible to block the glucocorticoid receptor (GR) mediated effects in experimental animals. To examine directly whether the effects of Dex on body fluid control were GR mediated, we used RU486 to determine if the effects of Dex on body fluid control were depended on activation of the GR. RU486 at a dose of 100 mg/kg completely reversed the effect of 24-hour Dex pretreatment on urinary excretion and water intake, and RU486 alone did not affect water intake and diuresis (Fig. 6A, B) in water-deprived rats. RU486 also prevented Dex-induced changes in NPR-A expression and cGMP levels in the hypothalamus (Fig. 6C, D, E).The antagonism of the effects of Dex by RU486 at 100 times concentration is consistent with previous results demonstrating RU486 inhibition of known GR receptor-mediated effects [17], [18], [19].

Anti-hANP antiserum (North Institute of Biological Technology, Beijing), which can neutralize endogenous ANP, was administrated intracerebroventricularly to inhibit the activation of hypothalamic NPR-A. Pretreatment with 2 µL anti-hANP antiserum (1∶10 dilution) was administrated intracerebroventricularly and blocked the inhibitory effect of glucocorticoids on water intake (Fig. 6F), which was consistent with previous findings that anti-ANP antiserum could potentiate dehydration or angiotensin II induced water intake in the rat [20], [21].

### Potent volume-depleting effect by Dex in rats given ad libitum access to food and water

**Figure 7:**
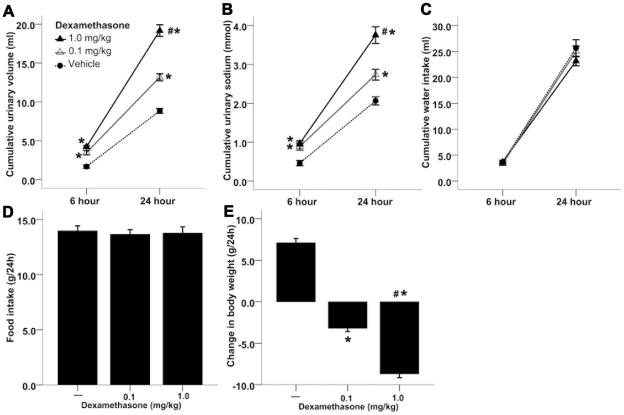
Potent volume depleting effect by Dex in rats given ad libitum access to food and water. [**A**] Effect of Dex on cumulative urinary volume; n = 10 for each group; * <0.01 compared with rats treated with vehicle; # <0.01 compared with rats treated with low dose of Dex. [**B**] Effect of Dex on cumulative urinary sodium; * <0.01 compared with rats treated with vehicle; # <0.01 compared with rats treated with low dose of Dex. [**C**] Effect of Dex on cumulative water intake. [**D**] Effect of Dex on food intake. [**E**] Effect of Dex on change from baseline in body weight; * <0.01 compared with rats treated with vehicle; # <0.01 compared with rats treated with low dose of Dex.

Thirty Wistar rats given ad libitum access to food and water were randomized to receive low dose of Dex (0.1 mg/kg), large dose of Dex (1 mg/kg) or vehicle. We monitored urinary volume, urinary sodium, water intake, food intake and body weight for 24 hours. Acute systemic glucocorticoid administration induced a potent renal water and sodium excretion (Fig. 7A, B). Of note, water intake and food intake remained the same between all three groups despite of the potent diuresis in Dex group (Fig. 7C, D), which led to a dramatic body weight reduction (i.e. systemic volume depletion) in the rats (Fig. 7E). Dex-treated animals did not drink more water than controls despite having much higher urine volumes.

## Discussion

Studies of glucocorticoids in body fluid metabolism have yielded conflicting results, which may reflect differences in dosage regimens, the time of treatment, or the specific body fluid volume status. The present study showed that glucocorticoids dramatically increased NPR-A density in the hypothalamus and an associated inhibitory effect on dehydration-induced water intake.

Many publications documented a paradoxical phenomenon that acute and chronic administration of glucocorticoid has different effects on renal water and sodium excretion. Acute administration of glucocorticoids produces diuresis and natriuresis, an effect more clearly demonstrable during the first 12 hours of administration, while chronic administration of the glucocorticoid attenuates renal water and sodium excretion [11], [12]. Our findings provided a clear answer for this phenomenon. Glucocorticoids increase renal and hypothalamic NPR-A density. Thus, they can produce potent diuretic effects and centrally inhibit water intake at the same time. It explained the phenomenon that glucocorticoids, such as Dex, could greatly increase urinary excretion of water without compensatory increases in water drinking in rats on ad libitum water and food at a short period, leading to dramatic volume depletion. The inhibitory effect of glucocorticoids on water intake increases with time, leading to progressive systemic volume depletion. Systemic volume depletion, in turn, compromises the ability of the kidney to excrete water and sodium. Nevertheless, the cumulative effects of chronic glucocorticoid treatment on water and sodium intake produce a negative balance of both substances [11]. This postulation is supported by the findings that central inhibition of endogenous ANP in the brain could increase spontaneous water intake, resulting in a dramatic increase in diuresis [21].

Because rats drink when they eat and eat when they drink, restriction of water or food effectively restricts consumption of both. It is well established that high doses of Dex inhibit food intake starting on the second day of treatment, that is, approximately 24 hours after Dex administration [22], [23]. Our study revealed a new aspect of glucocorticoids as a molecule regulating water intake. Thus, the inhibition of water intake may be part of a larger, or more general, phenomenon and may not be specific to water intake. Newly emerged evidence showed that ANP also played a role in modulating feeding activity patterns in rats [24]. A study by Tarjan showed intracerebral administration ANP not only inhibited water and salt intake, but reduced food intake, suggesting ANP may centrally inhibit food intake [25]. Further analyses on precise physiological implication of hypothalamic NPR-A activation between food intake and body fluid metabolism would lead to the better understanding of the significance of the natriuretic peptide system in water intake and food intake.

There is a widespread assumption that cortisol raises blood pressure as a consequence of renal water and sodium retention [26]. Given the fact glucocorticoids have potent volume-depleting effect, what are the mechanisms underlying glucocorticoid-induced hypertension? Previous researches found glucocorticoid-induced hypertension involves the endothelial nitric oxide system, which includes inhibition of inducible nitric oxide synthases and endothelial nitric oxide synthases isoforms, and inhibition of transmembrane arginine transport [27], [28], [29]. Furthermore, the natural ligand cortisol has plenty of mineralocorticoid activity, while the synthetic such as Dex is exclusively glucocorticoid in its effects. Therefore, the blood pressure response to glucocorticoids is more likely part of an overall mechanism to maintain hemodynamic homeostasis in the face of volume depletion.

Collectively, the data presented here, coupled with the ability of glucocorticoids to produce potent diuretic action in the fluid retention status [9], [30], [31], [32], [33], suggested that glucocorticoids played a central role in fluid volume control not only by stimulating potent diuretic action through activating renal natriuretic peptide system in the kidney, but also by blocking the neurogenic mechanisms responsible for the stimulation of water intake.

## Methods

### Ethics Statement

All animals were managed in accordance with the guidelines of Hebei Medical University, and the position of the American Heart Association on Research Animal Use. Experimental protocols were approved by the Institutional Animal Care and Use Committee of Hebei Medical University (approval ID: HebMU 20080301).

### Animal protocol

Male Wistar rats (provided by Hebei Medical University) weighing 220–250 g were housed in a temperature-controlled environment, exposed to a 12∶12-hour light-dark cycle, and given ad libitum access to a commercial standard diet of rat chow and water. All rats were housed in individual metabolic cages for one week to adapt to the environment before initiation of the study protocols.

### Western blotting analysis

The hypothalamuses were dissected out following a previous method. Briefly, the brains were placed ventral side up, and 4-mm-thick coronal slices were cut caudal to the optic chiasm. The slices were then dissected laterally up to the hypothalamic sulci and dorsally up to the mammilothalamic tract. Western blotting measurements were performed using the method previously prescribed [34]. NPR-A antibody (ab70848) was provided by Abcam Inc. Protein bands were digitally analyzed with Quantity One 1-D Analysis Software (Bio-Rad). The densities were expressed as a relative value compared with the average density measured in the vehicle treated rats. Data were normalized to β-actin for the individual animals.

### Surgical procedure

The detailed procedure of intracerebroventricular (ICV) guide cannula was prescribed before[35]. In brief, rats were fitted with an ICV guide cannula that was stereotaxically implanted to terminate in the third ventricle and fixed to the skull with dental acrylic and three stainless-steel screws. The following stereotaxic coordinates were used: 1.0 mm posterior to bregma, 1.0 mm lateral to the sinus and angled toward the midline at 10, and 7.5 mm ventral from the dura. One week of recovery was allowed for daily food and fluid intake, as well as body weight, to return to pre-surgery levels before tests were initiated.

### Analysis of plasma ANP

Plasma ANP was measured by radioimmunoassay. Blood samples were collected into chilled plastic tubes containing EDTA (1.5 mg/ml) and aprotinin (500 KIU/ml). Blood samples were centrifuged at 4°C, and the plasma samples were separated and stored at −70°C until assayed using radioimmunoassay. One milliliter of plasma was extracted on a C_l8_ SEP Pak column. The cartridges were prewashed with 10 ml of methanol and 10 ml of 4% acetic acid. After the plasma was applied, the cartridges were washed three times with 5 ml 0.1% vol/vol trifluoroacetic acid and the absorbed peptide was eluted with 2 ml 60% actronitrile/0.1% trifluoroacetic acid into plastic tubes. The extracts were dried down and reconstituted in 1.0 ml buffer and measured by radioimmunoassay Kit (provided by Neurobiology Department of The Second Military Medical University).

### Hypothalamic ANP content

After decapitation and blood collection from the trunk as described above, the hypothalamic tissues were quickly removed and boiled for 5 min in 10 volumes of 0.1 M acetic acid to abolish intrinsic proteolytic activity and then homogenized with a polytron homogenizer. The homogenates were centrifuged at 4000 rpm at 4°C for 20 min. The supernatant was lyophilized and stored at −20°C until assayed for ANP. Each sample was reconstituted with 1 ml of ANP buffer. Tissue ANP was expressed as per hypothalamus.

#### Immunofluorescence

Hypothalamic NPR-A expression was visualized by the indirect immunohistofluorescence method of Coons and coworkers[36]. Wistar rats were perfused through the heart first for 10 sec with ice-cold normal saline and then for 5–10 min with ice-cold phosphate-buffered 4% depolymerized paraformaldehyde solution. Brains were postfixed for 90 min in this perfusate and then soaked for at least 24 hours in 7% sucrose/0.6 M phosphate buffer, pH 7.4. Cryostat sections (14 µm thick) of hypothalamic region containing the median eminence were processed for the indirect immunofluorescence technique. Sections were stained at 37°C for 30 min with primary antibody (ab14356, Abcam Inc) diluted 1∶30 with phosphate-buffered saline containing 0.2% Triton X-100. The sections were then incubated for 18–24 hours at 4°C with the primary antibody. After three 5-min washes with phosphate-buffered saline/0.05% Triton, the sections were exposed for 3 hours at 37°C to fluorescein-conjugated guinea goat antibody against rabbit IgG (Goldenbridge biotechnology co., ltd) diluted 1∶50 with phosphate-buffered saline/0.1% Triton. The sections were then washed three times (5 min each) in phosphate-buffered saline/0.2% Triton, dipped in H_2_O, and mounted with 0.5 M sodium bicarbonate buffer (pH 8.4) diluted with an equal volume of glycerol. Each section was examined under darkfield conditions with a Nikon ECLIPSE 80i fluorescence microscope.

### Hypothalamic cGMP measurement

Hypothalamic tissues were homogenated in 1 ml Tris (0.05 mol/L) +4 mmol/L EDTA at 4°C, and the homogenates were heated for 3 min in a boiling water bath to coagulate proteins. After 20-min centrifugation at 4,000 rpm at 4°C, the supernatants were then assayed using cGMP radioimmunoassay kit provided by Shanghai University of T.C.M.

### Statistical analyses

All the data were express as means ± standard error of mean (s.e.m). Data in water intakes from the dehydration studies were analyzed by two-way repeated measures ANOVA, followed by a Student-Newman-Keuls test for multiple comparisons. Other statistical calculations were performed using a standard t-test of variables with 95% confidence intervals, unless otherwise stated.
